# Demulsification of Heavy Oil-in-Water Emulsion by a Novel Janus Graphene Oxide Nanosheet: Experiments and Molecular Dynamic Simulations

**DOI:** 10.3390/molecules27072191

**Published:** 2022-03-28

**Authors:** Yingbiao Xu, Yefei Wang, Tingyi Wang, Lingyu Zhang, Mingming Xu, Han Jia

**Affiliations:** 1 Key Laboratory of Unconventional Oil & Gas Development, China University of Petroleum (East China), Ministry of Education, Qingdao 266580, China; xuyingbiao.slyt@sinopec.com; 2 Technology Inspection Center, Shengli Oilfield Company, SINOPEC, Dongying 257000, China; wangtingyi180.slyt@sinopec.com (T.W.); zhangly639.slyt@sinopec.com (L.Z.); xumingming.slyt@sinopec.com (M.X.)

**Keywords:** heavy oil-in-water emulsion, demulsification, Janus graphene oxide, molecular dynamic simulation

## Abstract

Various nanoparticles have been applied as chemical demulsifiers to separate the crude-oil-in-water emulsion in the petroleum industry, including graphene oxide (GO). In this study, the Janus amphiphilic graphene oxide (JGO) was prepared by asymmetrical chemical modification on one side of the GO surface with n-octylamine. The JGO structure was verified by Fourier-transform infrared spectra (FTIR), transmission electron microscopy (TEM), and contact angle measurements. Compared with GO, JGO showed a superior ability to break the heavy oil-in-water emulsion with a demulsification efficiency reaching up to 98.25% at the optimal concentration (40 mg/L). The effects of pH and temperature on the JGO’s demulsification efficiency were also investigated. Based on the results of interfacial dilatational rheology measurement and molecular dynamic simulation, it was speculated that the intensive interaction between JGO and asphaltenes should be responsible for the excellent demulsification performance of JGO. This work not only provided a potential high-performance demulsifier for the separation of crude-oil-in-water emulsion, but also proposed novel insights to the mechanism of GO-based demulsifiers.

## 1. Introduction

The treatment of liquids produced from crude oil production has become a great challenge in the petroleum industry [1,2,3,4,5,6,7]. With excessive exploitation, most oilfields have reached the high water cut stage, a circumstance in which crude-oil-in-water emulsion was usually generated in the produced liquids [8,9]. In the case of heavy oil production, the ubiquitous asphaltene acts as natural emulsifier to readily adsorb on the oil/water interface and dramatically enhance the emulsion stability [10,11,12]. The extremely stable emulsion can cause serious problems to the downstream process, such as the generation of large amounts of polluting oily wastewater [13,14]. Therefore, it is an urgent issue to develop a highly efficient demulsifier to separate the oil from the emulsion generated in heavy oil exploitation.

The proposed strategies for oil–water separation mainly include adsorption [15], coalescence technology [16], membrane separation [17], gravity separation [18], flotation [19], and ultra-centrifugation [20]. Among these methods, chemical demulsification is widely applied owing to the tunable structure of demulsifier, high efficiency, and convenient operation [21]. Various chemical demulsifiers have been developed in last few decades, such as block copolymer [22], polysiloxane [23], hyperbranched polymer [24], dendrimer [25], and ionic liquids [26]. Chemical demulsifiers are widely used to break water-in-oil emulsions during the early stage of oilfield development. With the continuous development of oilfields, the study of oil-in-water emulsions has attracted a great deal of attention. While the increasing concern on environmental protection restricts the practical application of these chemicals [27], the research and development of novel demulsifiers is still a challenging and study-worthy topic for petroleum engineers.

Recently, nanomaterials have been regarded as another crucial category of chemical demulsifiers [28]. Nikkhah et al., verified that the settling time of nano-titania was significantly less than conventional chemical demulsifiers [29]. Zhang et al., fabricated the cyclodextrin-modified magnetic composite particles (M-CD) and evaluated its demulsification performance. M-CD could effectively separate various types of emulsions and exhibit excellent recycling ability [30]. Ren’s group systematically studied the demulsification performance of graphene oxide (GO) and its derivatives [31,32,33]. They concluded that the asphaltenes could effectively stabilize the oil-in-water emulsions under certain conditions, which created a large number of problems in practical production of the oilfield, and they proposed that the amphiphilic GO-based materials could adsorb on the oil/water interface to promote the coalescence of emulsion droplets.

It is well accepted that the amphiphilicity of GO is relatively weak due to its high content of hydrophilic oxygen-containing groups [34], while these active groups also provide the possibility for chemically hydrophobic modification of GO to improve its interfacial activity [35]. Janus graphene oxide (JGO) refers to GO with one side modified and has been applied in various fields due to its unique structure and properties [36,37]. For the petroleum industry, Luo et al., reported the application of JGO as a promising nano-fluid for enhanced oil recovery [38]. JGO with improved amphiphilicity can easily migrate to oil/water interface and interact with asphaltene, which may exhibit a better demulsification performance than GO.

Today, molecular dynamics (MD) simulations are employed extensively in the petroleum industry, which can reveal some interfacial properties of multiphase systems at the molecular level [39,40,41,42,43,44,45,46]. Stephan et al., investigated the vapor–liquid interface properties of binary mixtures (cyclohexane + CO_2_) via MD simulations [47]. Chakraborty et al., used MD simulations to study the vapor–liquid interface properties of n-heptane/nitrogen at different temperatures and pressures [48]. Lian et al., discussed the interaction of zwitterionic surfactant with various components of crude oil (asphaltene, resin, saturate, and aromatic) at the molecular level [49]. Liu et al., researched the emulsification and demulsification capabilities of a gas switchable surfactant through molecular dynamics simulations [50].

In this study, the JGO was synthesized by grafting n-octylamine on one surface of GO, and the successful modification was verified by FTIR, TEM, and contact angle measurements. Then, the demulsification efficiency of JGO was systematically investigated and compared with GO, including the effects of dosage, pH, and temperature. Based on the effects of JGO on the interfacial rheological properties and molecular dynamic (MD) simulation on the interaction between JGO and asphaltene, the passible demulsification mechanism of JGO was proposed.

## 2. Results and Discussion

### 2.1. Characterization of JGO

**Figure 1:**
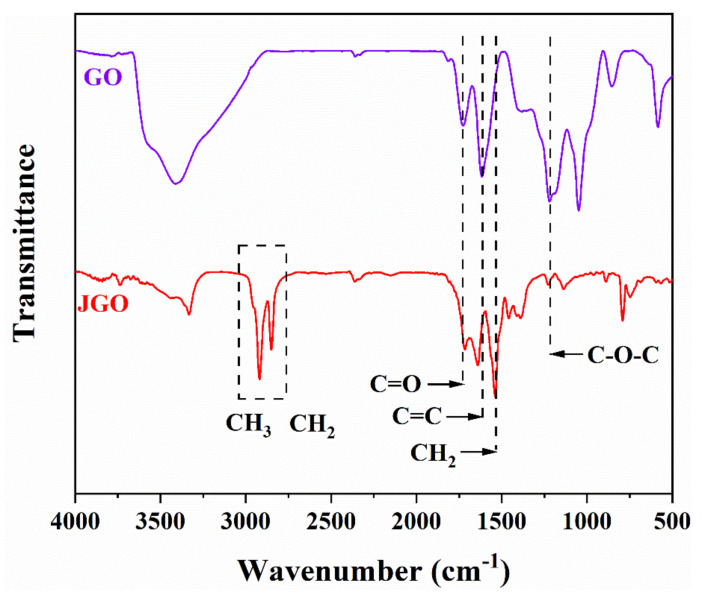
FTIR spectra of GO and JGO.

The FTIR spectra of GO and JGO were measured to verify the conjunction of n-octylamine on the JGO surface (Figure 1). The additional peaks between 3100 and 2800 cm^−1^ for the JGO sample were assigned to the stretching vibration of C-H (-CH_2_- and -CH_3_), which directly indicates the existence of n-octylamine with the hydrocarbon chain. Moreover, the weaker peaks at 1730 cm^−1^ (C=O) and 1208 cm^−1^ (C-O-C) in JGO’s curve than those in GO’s curve reflect that the n-octylamine was successfully grafted on the JGO surface via the reaction between amido group and epoxy/carboxyl groups.

**Figure 2:**
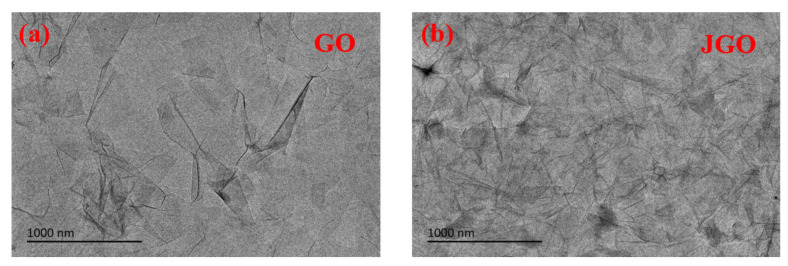
TEM images of GO (**a**) and JGO (**b**).

Figure 2 presents the TEM images of GO and JGO. It is obvious that the modified n-octylamine hardly changed the morphology and size of GO-based nanosheets, but only resulted in more overlapped parts in JGO due to the additional hydrophobic interaction. To further demonstrate the Janus structure of JGO, the JGO interfacial film constructed at the octane/water interface was transferred to the glass substrate to measure water contact angles of both sides of the JGO. As shown in Figure S1, the unmodified side of JGO was relatively hydrophilic with a water contact angle of 32°, which was attributed to the abundant oxygen-containing groups. For the n-octylamine grafted side of JGO, the water contact angle increased to 85° due to the hydrophobic hydrocarbon chain. The opposite affinity to water for both sides of JGO directly confirmed the generation of the Janus structure.

### 2.2. Demulsification Efficiency Tests

**Figure 3:**
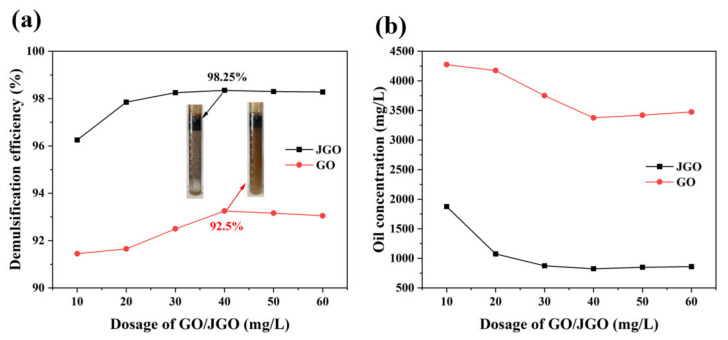
The demulsification efficiency (**a**) and oil concentrations (**b**) of GO and JGO with different dosages.

The demulsification efficiency of GO and JGO for the stable heavy oil-in-water emulsions (prepared with 5% heavy oil) was evaluated by the bottle test (Figure 3). The blank sample exhibited excellent stability, with almost no separated oil from the emulsion (Figure S2). The optimal demulsification efficiency of JGO was achieved at the much lower concentration (40 mg/L), and the oil content was sharply reduced to 825 mg/L. The emulsion color became lighter with additional demulsifiers (GO or JGO), and the emulsion droplets spontaneously aggregated to form a separated oil layer. Meanwhile, there were flocculent aggregates on the top of the water phase. The higher demulsification efficiency of JGO (98.25%) than that of GO (92.5%) indicates that the asymmetrically modified n-octylamine can greatly improve the demulsification performance of GO-based nanosheets. Higher concentrations of JGO slightly decreased the demulsification efficiency, which may be ascribed to the adsorbed oil on the JGO surface.

**Figure 4:**
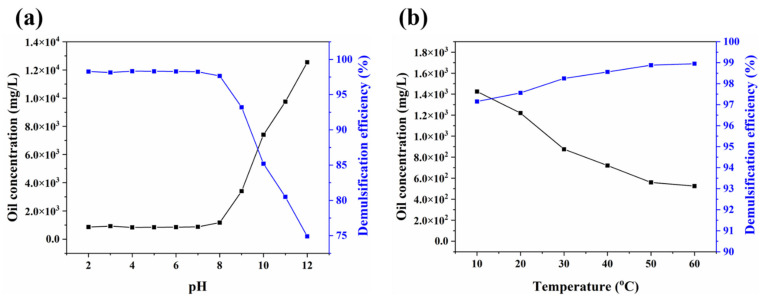
The effects of pH (**a**) and temperature (**b**) on the demulsification performance of JGO (30 mg/L).

To systematically investigate the demulsification performances of JGO (30 mg/L) under different conditions, the effects of pH and temperature were studied via a series of experiments (Figure 4). It is well known that the solution pH value can significantly affect the surface physiochemical properties of the demulsifiers to impact their performances [27]. For JGO, the demulsification efficiency at acidic and natural conditions was excellent, but the alkaline environment was adverse to its performance (Figure 4a). On the one hand, the organic acid in crude oil would react with alkali to generate a surface-active substance, which could largely improve the emulsion stability. On the other hand, the variation of zeta potential of JGO and emulsions at different pH values should be the other factor for the pH-dependent demulsification efficiency of JGO (Figure S3). With increasing pH values, both JGO and emulsion droplets became more negatively charged. Therefore, the intensive electrostatic repulsion between JGO and emulsion droplets at the alkaline condition (pH ≧ 8) would weaken the demulsification efficiency of JGO. In addition, the demulsification efficiency of JGO slightly increased with increased temperature (Figure 4b). Although the higher temperature may disturb the interaction between JGO and emulsion droplets, the accelerated thermal movements of emulsion droplets facilitated their coalescence. More importantly, it was verified that JGO can effectively separate heavy oil-in-water emulsions in a wide temperature range.

### 2.3. Demulsification Mechanism of JGO

**Figure 5:**
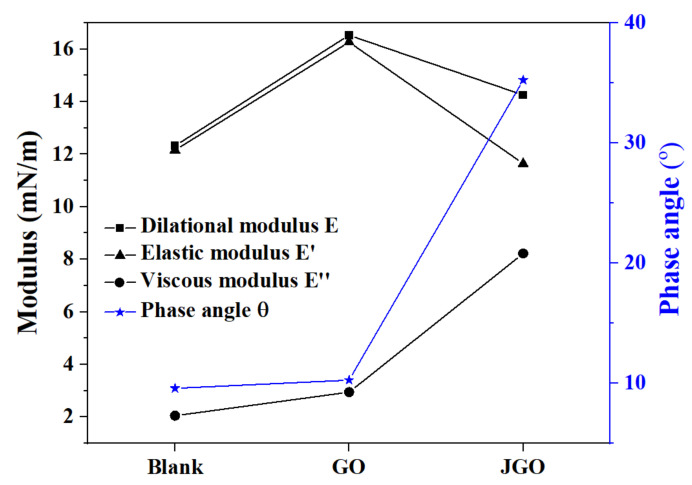
Effects of GO and JGO on the interfacial rheology of crude oil/water interface.

For the emulsions prepared in this study, the emulsion stability is mainly dependent on the surface-active substance in crude oil, asphaltene in particular. Asphaltene could fabricate a protective film at the oil/water interface to prevent the coalescence of the emulsion droplets [31]. Therefore, the additional demulsifier should primarily destroy the asphaltene film to achieve demulsification. To investigate the effects of GO and JGO on the interfacial properties of the crude oil/water interface, the interfacial dilatational rheology experiment was conducted. As shown in Figure 5, the dilatational modulus of crude oil/water system was 12.32 mN/m, which should be attributed to the interfacial adsorption of asphaltene. The addition of GO or JGO in the water phase causes the evident increase of the dilatational modulus in different degrees. GO with the hydrophilic edge and the hydrophobic plane is generally regarded as the amphiphile [51], whereas the amphiphilicity of JGO was further improved by the asymmetric modification with n-octylamine. Then, the interfacial film is the combination of asphaltene and GO or JGO. For the blank and GO system, the interfacial film was basically elastic, with the phase angle around 10°, while the phase angle of JGO system increased dramatically, indicating the typical viscoelastic property of the interfacial film. The smaller *E* and larger *E″* in the JGO system could be ascribed to the Janus structure. Considering GO and JGO nanosheets are 2D materials with an average literal size around 500 nm, the adsorbed GO or JGO at the interface should be overlapped with each other. The overlapped parts of JGO nanosheets may not be as rigid as those of GO nanosheets due to the hinderance of n-octylamine. Therefore, the JGO adsorbed interface of the emulsion would be much easier to deform, leading to the lower *E* and more distinct viscous characteristic.

**Figure 6:**
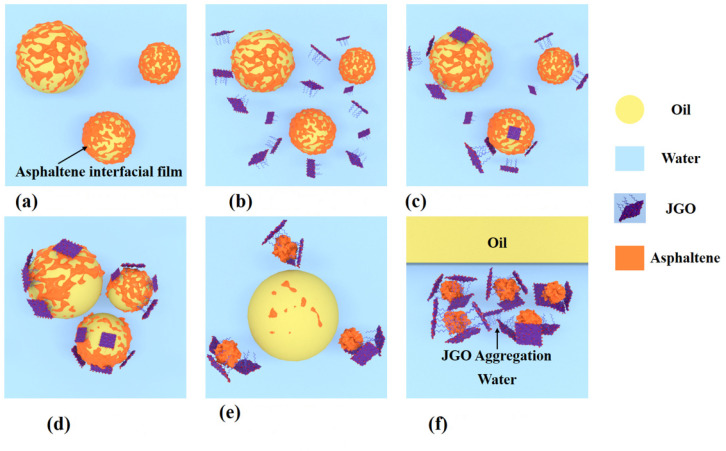
Schematic of the demulsification mechanism of JGO; (**a**) stable emulsion; (**b**) adding JGO; (**c**) adsorption of JGO; (**d**) shaking; (**e**) aggregation of oil droplets; (**f**) after settling.

Based on the results of interfacial rheology, the possible demulsification mechanism of JGO could be proposed (Figure 6). The adsorbed asphaltene molecules constructed the protective film at the oil/water interface, generating a new phase to stabilize the heavy oil-in-water emulsion from the thermodynamic aspect (Figure 6a) [41,52]. After JGO was added to the emulsion system, it could disperse well in the water phase (Figure 6b). Then, JGO with improved amphiphilicity could adsorb at the oil/water interface to interact with asphaltene (Figure 6c). During the shaking, the emulsion droplets collided with each other (Figure 6d). The intensive interaction between the amphiphilic JGO and the surface-active substance asphaltene could destroy the original interfacial film, and parts of asphaltene desorbed from the oil/water interface, leading to the coalescence of smaller ones without the protective film (Figure 6e). Finally, after settling, the larger oil droplets moved upward to form a separated oil layer, and the JGO/asphaltene aggregation also moved to the upper region of the water phase due to the highly improved hydrophobicity (Figure 3a and Figure 6f). The greater amphiphilicity of JGO should be responsible for its higher demulsification efficiency than GO.

### 2.4. Verification of the Mechanism via Molecular Dynamic Simulation

**Figure 7:**
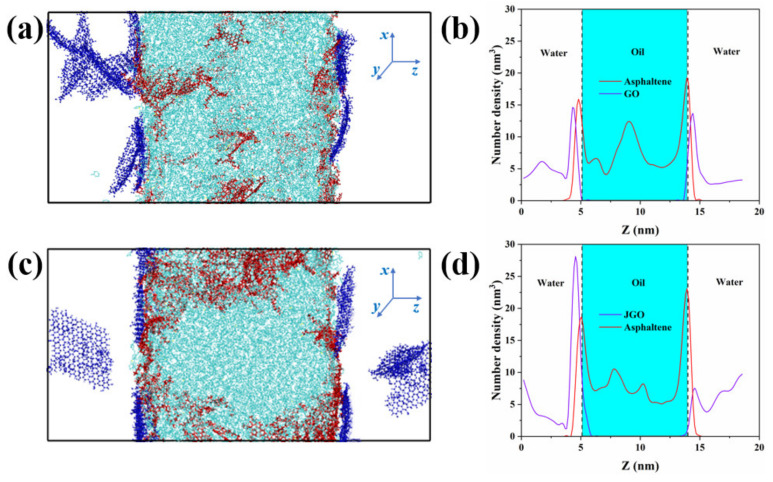
Final simulation configuration (at 50 ns) of GO (**a**) and JGO (**c**) in the oil/water system. Oil components are colored in cyan except asphaltene which is colored in red, and GO and JGO are colored in blue. Water molecules are not shown for clarity. Number density of three asphaltene in oil, GO (**b**), and JGO (**d**) along the z-direction. Gray dash lines in (**b**,**d**) represent the Gibbs partition surface.

To verify the mechanism of the JGO as a high-performance demulsifier, the behavior of GO and JGO in the crude oil/water system was simulated via molecular dynamic (MD) simulation. As shown in Figure S4, GO and JGO nanosheets were randomly placed in the water phase in the initial configuration, which could achieve the dynamic equilibrium after 50 ns MD simulation (Figure S5). The Gibbs partition surfaces of two systems were located at ~5.1 nm and ~14.0 nm, and the density peaks of asphaltene (including asphaltene-1, asphaltene-2, and asphaltene-3 in Scheme S1), GO, and JGO in two systems were all around the Gibbs partition surface, indicating that the GO-based nanosheets can spontaneously adsorb at the crude oil/water interface and interact with asphaltene (Figure 7). Compared with GO, there was more JGO adsorbed at the interface with the n-octylamine modified side toward the oil phase. Meanwhile, the density peak of asphaltene in the JGO system was much greater than that in the GO system.

**Figure 8:**
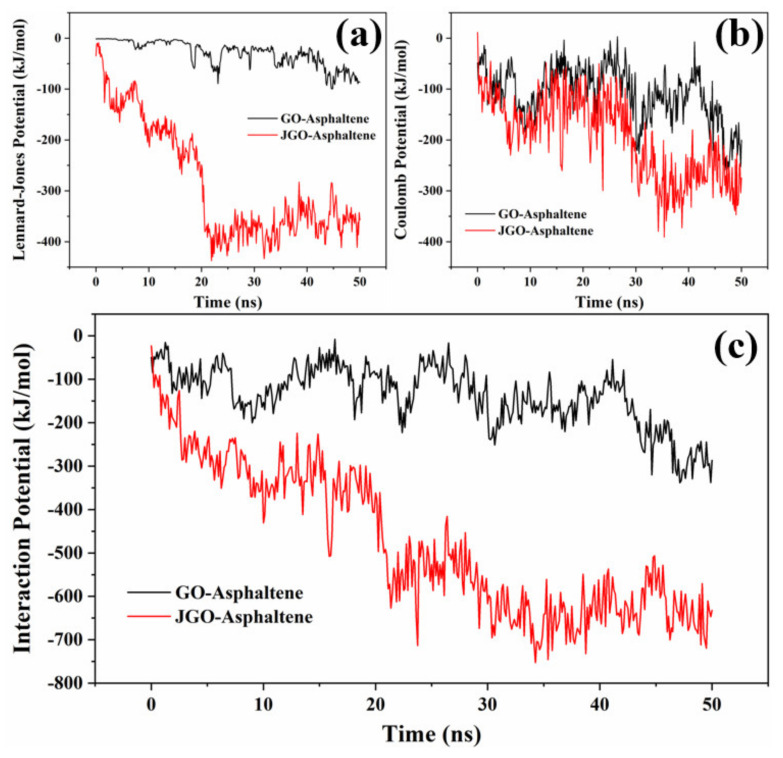
Intermolecular interaction energy between GO/JGO and asphaltene at the oil/water interface: Lennard–Jones potential (**a**), Coulomb potential (**b**), and total interaction energy (**c**).

To quantificationally investigate the interaction energy between GO/JGO and asphaltene, the nonbonded interaction, including Lennard–Jones potential and Coulomb potential, during the whole simulation process was extracted (Figure 8a,b). The much larger total interaction energy between JGO and asphaltene (~−650 kJ/mol) than that between GO and asphaltene (~−250 kJ/mol) further confirmed their more intensive interaction (Figure 8c). It is worth noting that the much larger interaction energy in the JGO-asphaltene system was mainly derived from the stronger Lennard–Jones potential, which should be ascribed to the hydrophobic interaction among the alkyl chains on both JGO and asphaltene.

**Figure 9:**
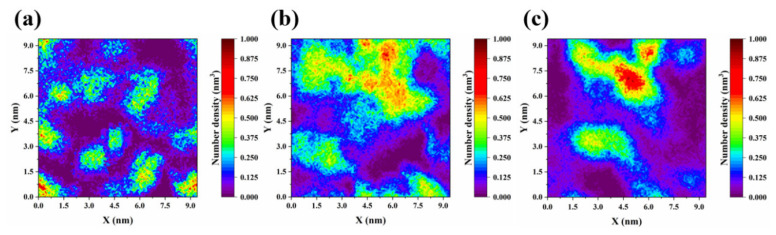
Two-dimensional number density maps of asphaltene distribution in the XY plane at the oil/water interface in the GO-free (**a**), GO (**b**), and JGO (**c**) systems.

As mentioned in the proposed mechanism, the asphaltene-constructed protective film was essential for the emulsion stability. Then, the distribution characteristic of asphaltene at the crude oil/water interface was further analyzed by calculating and plotting the two-dimensional (2D) number density map in the XY plane (Figure 9). The color distribution of the 2D number density maps directly indicates the asphaltene distribution and compactness in the GO-free, GO, and JGO systems. In the GO-free system, the more green and less yellow/red areas reflect the uniform distribution of asphaltene at the crude oil/water interface (Figure 9a). Compared with that in the GO system, the generation of dark red and red areas represents the much greater asphaltene density at the local interface (Figure 9b,c). On the one hand, the uneven distribution of asphaltene should be attributed to the constraint of the intensive interaction between JGO and asphaltene. On the other hand, the uneven distribution of asphaltene may cause the evident weakness in the protective film, resulting in the easy coalescence of the emulsion droplets.

## 3. Experimental

### 3.1. Materials

Graphene oxide (GO) was purchased from Shengzhen Turing Evolution Technical Company, China. Paraffin wax (with a melting point around 58−60 °C), kerosene, n-octylamine (>98%), ethanol (>99.5%), n-octane (>99%), and NaCl (>99.5%) were products from Shanghai Aladdin Biochemical Technology Co., Ltd., Shanghai, China.

### 3.2. Preparation of JGO

The method applied for the preparation of JGO was reported by Luo et al. [53]. First, 200 mL GO aqueous solution (1 mg/mL), 6 g NaCl, and 50 g paraffin wax were heated to 75 °C and stirred with a homogenizer at 10,000 rpm for 10 min to form emulsions, which were cooled to ambient temperature and filtered to obtain GO-coated wax particles. After the GO-coated wax particles were washed by NaOH solution (pH ~10), deionized (DI) water, and ethanol successively, the wax particles were added into the n-octylamine ethanol solution (0.4489 mg/mL) and magnetically stirred overnight. Then, the wax particles were washed with ethanol and dissolved in toluene to remove the wax, and JGO was obtained by centrifugation. Finally, JGO was dried at 60 °C and then dispersed in DI water at certain concentrations.

### 3.3. Characterization

The Fourier-transform infrared spectra (FTIR) of GO and JGO were recorded by a PerkinElmer Spectrum Two spectrometer (PerkinElmer, Waltham, MA, USA). Transmission electron microscopy (TEM) images of GO and JGO were obtained with a JEOL JEM-1400 transmission electron microscope (JEOL, Tokyo, Japan).

The contact angle measurements were conducted to the affinity of both sides of JGO. First, the interfacial film was fabricated by shaking the glass tube filled with JGO aqueous solution and n-octane, then the n-octane was removed by evaporation. To measure the contact angle of the n-octylamine modified (hydrophobic) side of JGO, the pre-cleaned glass slide was lifted below the JGO interfacial film to deposit the film on the glass substrate. To measure the contact angle of the unmodified (hydrophilic) side of JGO, the pre-cleaned glass substrate was pressed onto the interfacial film and rotated immediately. After the obtained glass substrates were dried at 40 °C, the water contact angle measurement was conducted with a contact angle goniometer (JC2000D5M, Zhongchen, China).

### 3.4. Demulsification Efficiency Test

The heavy oil used in this study was obtained from Shengli oil field. To prepare stable heavy oil-in-water emulsions, 5 g heavy oil and 95 g NaCl aqueous solution (3000 mg/L) were mixed at 60 °C and stirred with a FJ200-S homogenizer at 12,000 rpm for 10 min. For the demulsification test, a certain amount of JGO (or GO as control sample) aqueous solution was added into the prepared emulsion stored in the measuring cylinder. Then, the cylinder was shaken for 1 min to ensure the thorough mixture of JGO and the emulsion. Then, the cylinder was placed at ambient temperature and the oil/water separation process was recorded by a camera. The oil content in the water phase after demulsification process was determined by UV–Vis spectrophotometer. The standard curve of oil content as a function of absorbance at 256 nm was obtained by measuring the absorbance of a series of mixture with different oil content. The demulsification efficiency ($E_{D}$) was calculated by the following equation:

(1) $$E_{D}=\frac{C_{0}-C_{1}}{C_{0}}\times 100\%$$

where $C_{0}$ is the initial oil content before demulsification, and $C_{1}$ is the oil content after demulsification.

### 3.5. Interfacial Dilatational Rheology Measurement

The interfacial property was measured by a dynamic interfacial oscillatory drop tensionmeter (Tracker, Teclis, France). A drop of crude oil (20 μL) was injected into the solutions through an inverted needle, and the volume of the oil drop changed in sinusoidal oscillatory motion, which was achieved by a motor system connected to the syringe. Based on the shape variation of the oil drop recorded by a CDD digital camera, the IFT was calculated according to the Laplace–Young equation and the Plane hydrostatic equation by computer software. At the same time, the dilatational modulus (*E*) was calculated by the software using the following equation [54]:

(2) $$E=\frac{d\gamma }{dlnA}$$

where *γ* is the interfacial tension (mN/m), and *A* is the interface area (m^2^).

The dilatational modulus can also be expressed in plural form:

(3) $$E=E^{\prime }+E^{\prime \prime }=E^{\prime }+i\omega \eta_{d}$$

where *E′* is the elastic modulus, *E″* is the viscous modulus, *ω* is the interfacial dilatation frequency, and *η_d_* is the interfacial dilatation viscosity.

### 3.6. Simulation Method

#### 3.6.1. Construction of Crude Oil/Water System

The crude oil model in this simulation was constructed based on the typical heavy oil model, which is composed by asphaltene, resin, aromatic, and saturate components [55,56]. Based on previous literature, three typical asphaltenes molecules and six types of resin molecules were applied in this crude oil model [55,56]. The aromatic components mainly include toluene and benzene. As for saturates, cycloheptane, cyclohexane, nonane, octane, heptane, and hexane were incorporated. The detailed information of the crude oil components was listed in Table S1, and the molecular structures of asphaltene and resin were depicted in Scheme S1.

To obtain the crude oil model, all component molecules were added randomly into a cubic box (12 × 12 × 12 nm^3^), and 20 ns MD simulation under NPT ensembles was performed to achieve the reasonable density. The final crude oil model was obtained with the cubic box size of 9.4 × 9.4 × 9.4 nm^3^, and the box was extended along z-direction to 9.4 × 9.4 × 18.8 nm^3^. The empty parts of the extended crude oil box were filled with water molecules and counterions (Na^+^) to establish the initial oil/water interface. Finally, 50 ns MD simulation under NPT ensemble was carried out to achieve the dynamic equilibrium of crude oil/water simulation system.

#### 3.6.2. Construction of GO and JGO in Crude Oil/Water System

The single layer of GO was built with 286 carbon atoms as the substrate, 6 carboxyl groups on the edge, 25 epoxy groups on each side, 10 hydroxyl groups on each side, and 12 hydroxyl groups on the edge [57]. Based on the model of GO, six n-octylamine molecules were attached on one surface of GO via the ring-opening of epoxy groups to form JGO. The molecular structures of GO and JGO were illustrated in Scheme S2. Then, 10 GO/JGO were randomly inserted into the water phase of a pre-equilibrated crude oil/water system. The 50 ns MD simulation under NPT ensemble was carried out to achieve the dynamics equilibrium of the simulation system.

#### 3.6.3. MD Simulation Methods

The GROMACS (version 2019.6) software package was used to perform MD simulations with optimized potentials for liquids simulation-all atom (OPLS-AA) force field [58]. Molecular parameter sets were generated from the LigParGen web server [59]. The TIP4P model was adopted to describe the water molecule. The steepest descent method was used to minimize the simulation system, and the convergence criterion of energy minimization was 50 kJ/(mol·nm). Then, the MD simulation with NPT ensemble at 1 atm and 303 K was carried out for each system. In the simulation, the velocity rescaling thermostat and Berendsen (first 10 ns) + Parrinello-Rahman (last 40 ns) pressure coupling were employed as temperature coupling method and pressure coupling method, respectively [60,61,62]. The LINCS algorithm was used to constrain the bonds with H atoms [63]. The cut off scheme was applied in the van der Waals (vdW) interaction. Coulomb interaction was computed using the Particle-Mesh Ewald (PME) method [64]. The simulation time step was 2 fs and trajectories were saved every 10 ps for further analysis, which was visualized by visual molecular dynamics (VMD) software [65].

## 4. Conclusions

In conclusion, we successfully synthesized n-octylamine-modified JGO. Compared with GO, JGO exhibited superior ability to effectively separate the heavy oil-in-water emulsion with a demulsification efficiency as high as 98.25% at the much lower concentration (40 mg/L). The interfacial dilatational rheology measurements demonstrated that the additional JGO to heavy oil-in-water emulsion systems could improve the viscoelasticity of the elastic oil/water interface, which facilitated the deformation of the interface and the destruction of the protective film. Moreover, the MD simulation further verified the more intensive adsorption of JGO on the crude oil/water interface and the stronger interaction between JGO and asphaltene in comparison to the GO system. Therefore, it is believed that the remarkable demulsification ability of JGO should be attributed to its powerful attraction with asphaltene, leading to the easily deformable oil/water interface and the uneven distribution of asphaltene. This study indicates that JGO could be applied as a high-performance demulsifier to separate heavy oil-in-water emulsions in the oil industry, and the proposed mechanism of JGO could be inspiring for a new strategy of chemical demulsifier design.

## Data Availability

The data presented in this study are available on request from the corresponding authors.

## Supplementary Materials

The following supporting information can be downloaded at: https://www.mdpi.com/article/10.3390/molecules27072191/s1, Scheme S1: Molecular structures of different asphaltenes and resins; Scheme S2: Molecular structures of GO and JGO; Figure S1: The water contact angle of the unmodified side of JGO (a) and n-octylamine grafted side of JGO (b); Figure S2: Demulsification performance of GO and JGO; Figure S3: Zeta potential of JGO and oil droplets with increasing pH values; Figure S4: The initial configuration of GO and JGO in the crude oil/water system, oil components are colored in cyan excepting asphaltenes which are colored in red, and GO and JGO are colored in blue. Water molecules are not shown for clarity; Figure S5: Total energy curves in the simulation process with 10 GO (a) and JGO (b) randomly inserted into oil/water systems; Table S1: Compositions of the crude oil model.
