# Supplementary material for: Demulsification of Heavy Oil-in-Water Emulsion by a Novel Janus Graphene Oxide Nanosheet: Experiments and Molecular Dynamic Simulations
Source: Molecules. 2022 Mar 28;27(7):2191. doi: 10.3390/molecules27072191 (PMC9000454; doi:10.3390/molecules27072191)
Supplement: Supplementary file 1 [file molecules-27-02191-s001.zip › molecules-1635276 supplementary.pdf]

Supplementary Materials

Demulsification of Heavy Oil-In-Water Emulsion by a Novel Janus Graphene Oxide Nanosheet: Experiments and Molecular Dynamic Simulations

Yingbiao Xu [1,2], Yefei Wang [1,*], Tingyi Wang [2], Lingyu Zhang [2], Mingming Xu [2] and Han Jia [1,*]

[1] Key Laboratory of Unconventional Oil & Gas Development, China University of Petroleum (East China), Ministry of Education, Qingdao 266580, China; xuyingbiao.slyt@sinopec.com
[2] Technology Inspection Center, Shengli Oilfield Company, SINOPEC, Dongying 257000, China; wangtingyi180.slyt@sinopec.com (T.W.); zhangly639.slyt@sinopec.com (L.Z.); xumingming.slyt@sinopec.com (M.X.)
* Correspondence: wangyf@upc.edu.cn (Y.W.); jiahan@upc.edu.cn (H.J.)

Table S1. Compositions of the crude oil model.

Component	Number	Component	Number
Asphaltene-1	23	Toluene	359
Asphaltene-2	23	Benzene	138
Asphaltene-3	23	Cycloheptane	359
Resin-1	53	Cyclohexane	221
Resin-2	53	Nonane	414
Resin-3	53	Octane	359
Resin-4	53	Heptane	304
Resin-5	53	Hextane	331
Resin-6	53		

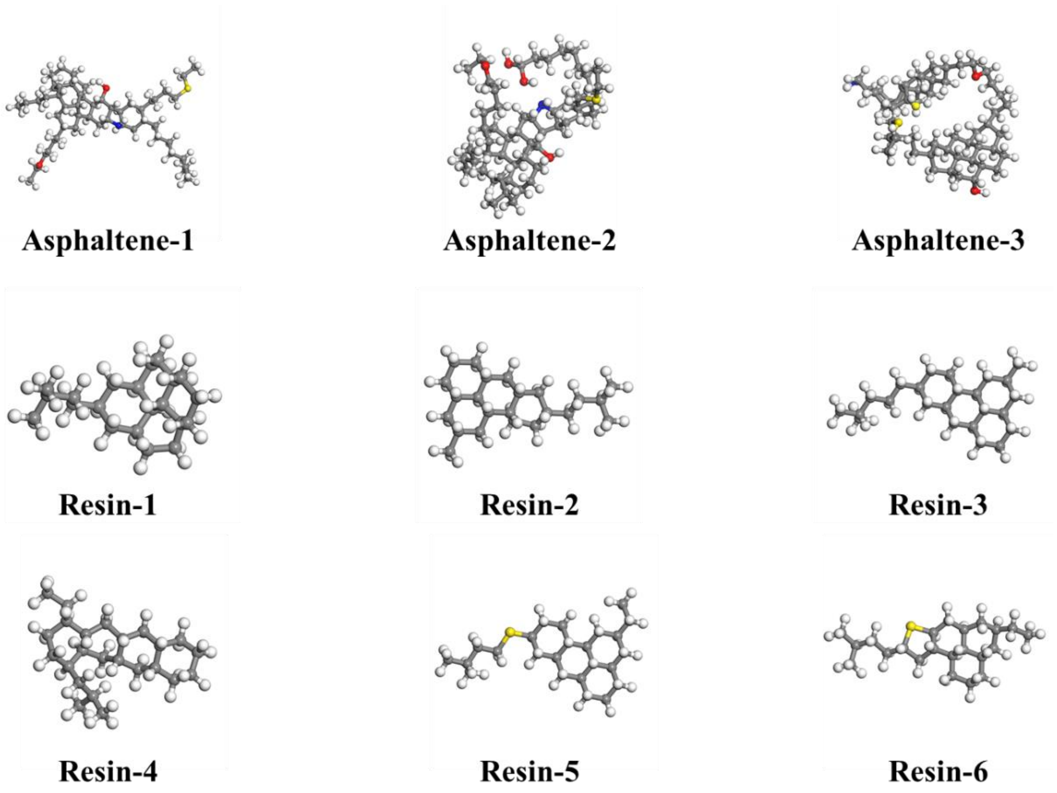

Scheme S1. Molecular structures of different asphaltenes and resins.

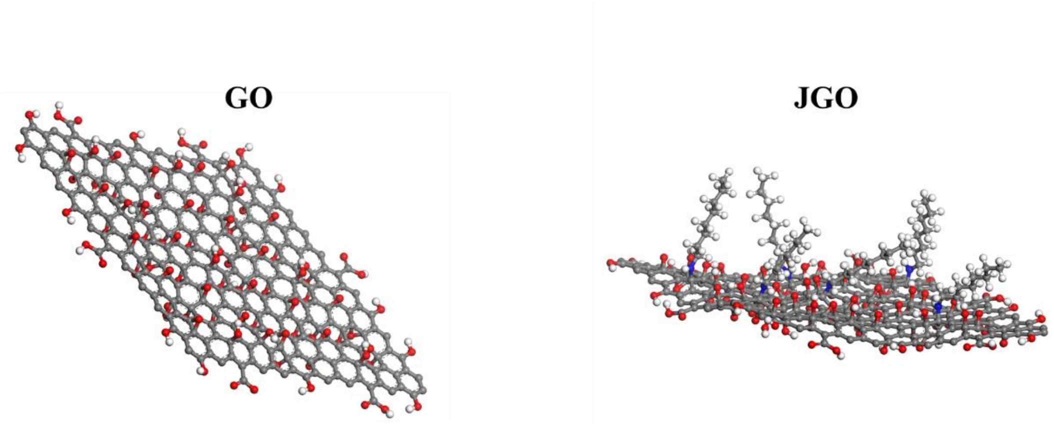

Scheme S2. Molecular structures of GO and JGO.

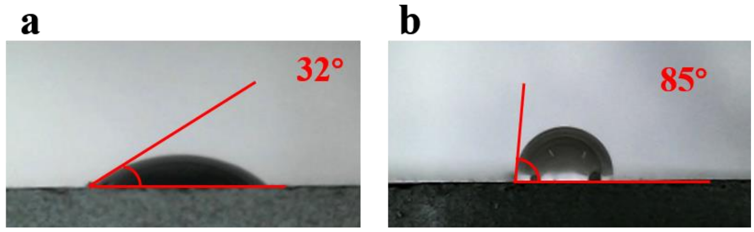

Figure S1. The water contact angle of the unmodified side of JGO (**a**) and n-octylamine grafted side of JGO (**b**).

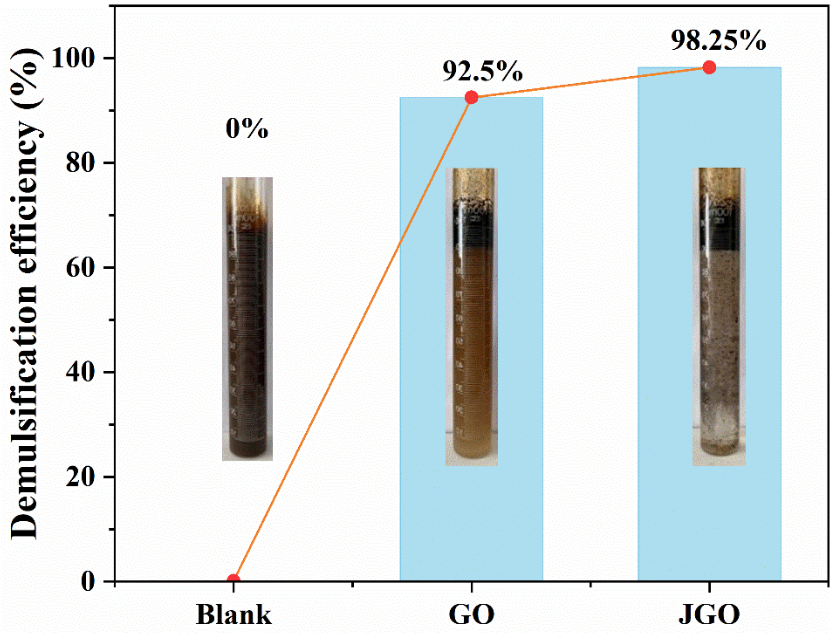

Figure S2. Demulsification performance of GO and JGO.

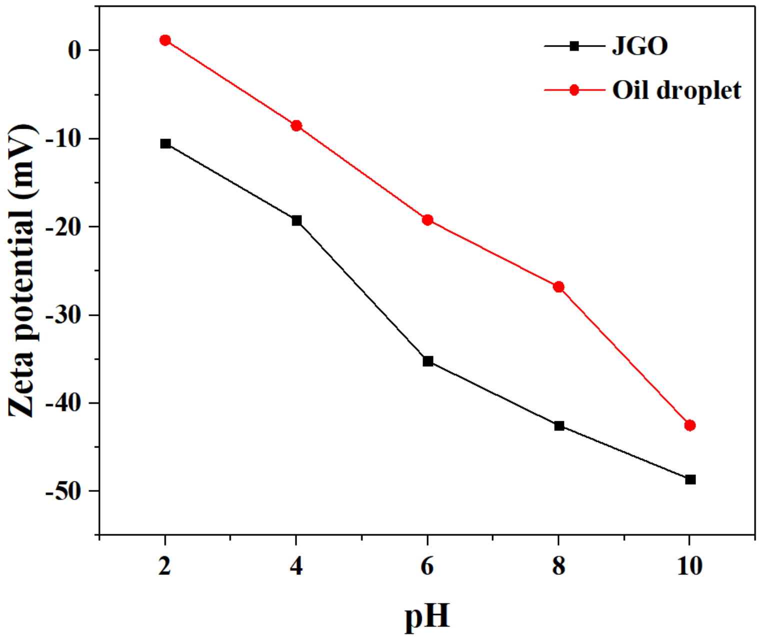

Figure S3. Zeta potential of JGO and oil droplets with increasing pH values.

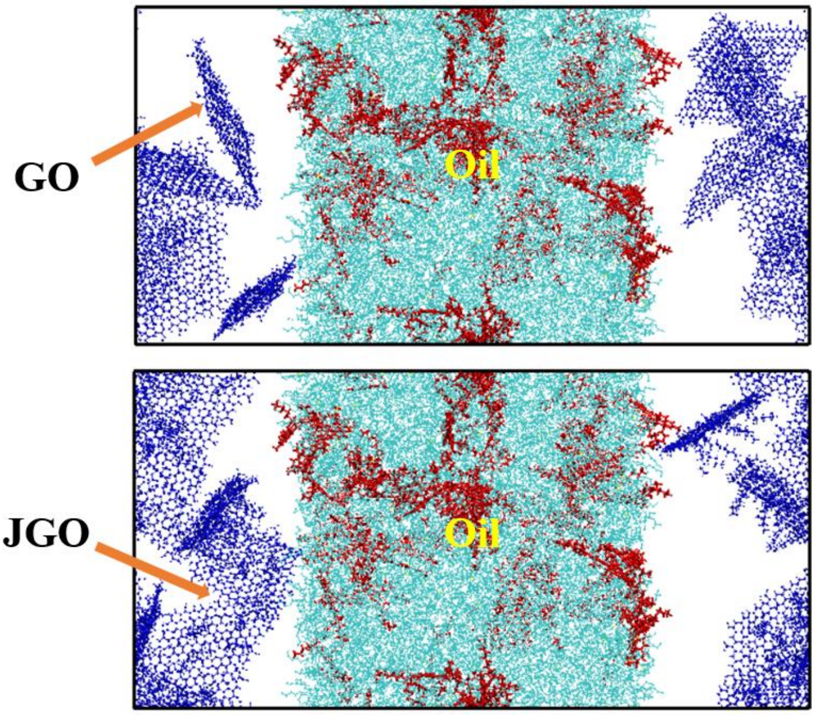

Figure S4. The initial configuration of GO and JGO in the crude oil/water system, oil components are colored in cyan excepting asphaltenes which are colored in red, and GO and JGO are colored in blue. Water molecules are not shown for clarity.

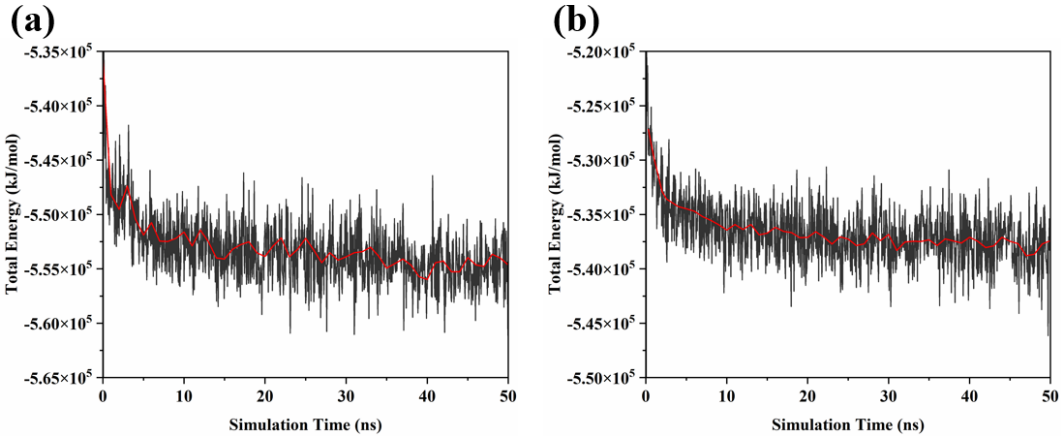

Figure S5. Total energy curves in the simulation process with 10 GO (**a**) and JGO (**b**) randomly inserted into oil/water systems.
